# Should heart age calculators be used alongside absolute cardiovascular disease risk assessment?

**DOI:** 10.1186/s12872-018-0760-1

**Published:** 2018-02-07

**Authors:** Carissa Bonner, Katy Bell, Jesse Jansen, Paul Glasziou, Les Irwig, Jenny Doust, Kirsten McCaffery

**Affiliations:** 1 Wiser Healthcare Program, Sydney School of Public Health, The University of Sydney, Camperdown, NSW 2006 Australia; 2 Centre for Medical Psychology and Evidence-based Decision-making (CeMPED), The University of Sydney, Camperdown, NSW 2006 Australia; 3 Faculty of Health Sciences and Medicine, Bond University, Robina, QLD 4229 Australia

**Keywords:** Cardiovascular risk, Risk assessment, Heart age, Overtreatment, Overdiagnosis

## Abstract

**Background:**

National estimates of ‘heart age’ by government health organisations in the US, UK and China show most people have an older heart age than current age. While most heart age calculators are promoted as a communication tool for lifestyle change, they may also be used to justify medication when clinical guidelines advocate their use alongside absolute risk assessment. However, only those at high absolute risk of a heart attack or stroke are likely to benefit from medication, and it is not always clear how heart age relates to absolute risk. This article aims to: 1) explain how heart age calculation methods relate to absolute risk guidelines; 2) summarise research investigating whether heart age improves risk communication; and 3) discuss implications for the use of medication and shared decision making in clinical practice.

**Main body:**

There is a large and growing number of heart age models and online calculators, but the clinical meaning of an older heart age result is highly variable. An older heart age result may indicate low, moderate or high absolute risk of a heart attack or stroke in the next 5-10 years, and the same individual may receive a younger or older heart age result depending on which calculator is used. Heart age may help doctors convey the need to change lifestyle, but it cannot help patients make an informed choice about medication to reduce CVD risk.

**Conclusion:**

Interactive heart age tools may be helpful as a communication tool to initiate lifestyle change to reduce risk factors. However, absolute risk should be used instead of heart age to enable informed decision making about medication, to avoid unnecessary treatment of low risk people. Evidence-based decision aids that improve patient understanding of absolute risk should be considered as alternatives to heart age calculators for lifestyle and medication decisions.

## Background

A recent newspaper front page in the UK exclaimed “4 in 5 have a heart that is older than they are”, with similar media reports in the US that “40% of Americans had hearts that were five or more years older than their actual ages” [1, 2]. These alarming statistics might suggest a need for mass medicalisation of the population with statins and blood pressure lowering drugs, but what does it really mean to have an older heart age? This article explores the idea that the heart age concept is highly variable and potentially misleading when it comes to deciding about whether or not to take preventive medication [3, 4]. This is in contrast to guidelines advocating the use of heart age alongside absolute risk assessment for medication decision making [5]. The absolute risk of a heart attack or stroke is a better way to enable informed decision making and target treatment to those at highest risk who are most likely to benefit; while at the same time avoiding unnecessary labelling and treatment of low risk people [6, 7]. There is increasing awareness that we might be overdiagnosing and overtreating healthy, asymptomatic people, and that ‘less is more’ when the harms of an intervention outweigh the benefits [8]. Concerns about ‘mass medicalisation’ for cardiovascular disease (CVD) prevention have already been voiced following new guidelines in the UK and the US advocating much lower absolute risk thresholds for medication: halved from 20% to 10% over 10 years in the UK; and even lower to 7.5% in the US [9, 10].

This article aims to: 1) provide an overview of heart age calculation methods used in connection to absolute risk guidelines; 2) summarise research investigating whether heart age improves risk communication; and 3) discuss implications for the use of medication and shared decision making in clinical practice. We argue that the increasingly popular concept of heart age may contribute to mass medicalisation if used for the wrong purpose, and provide directions for future research to explore alternative risk communication formats that enhance informed decision making.

## Main text

### What is heart age?

**Fig. 1:**
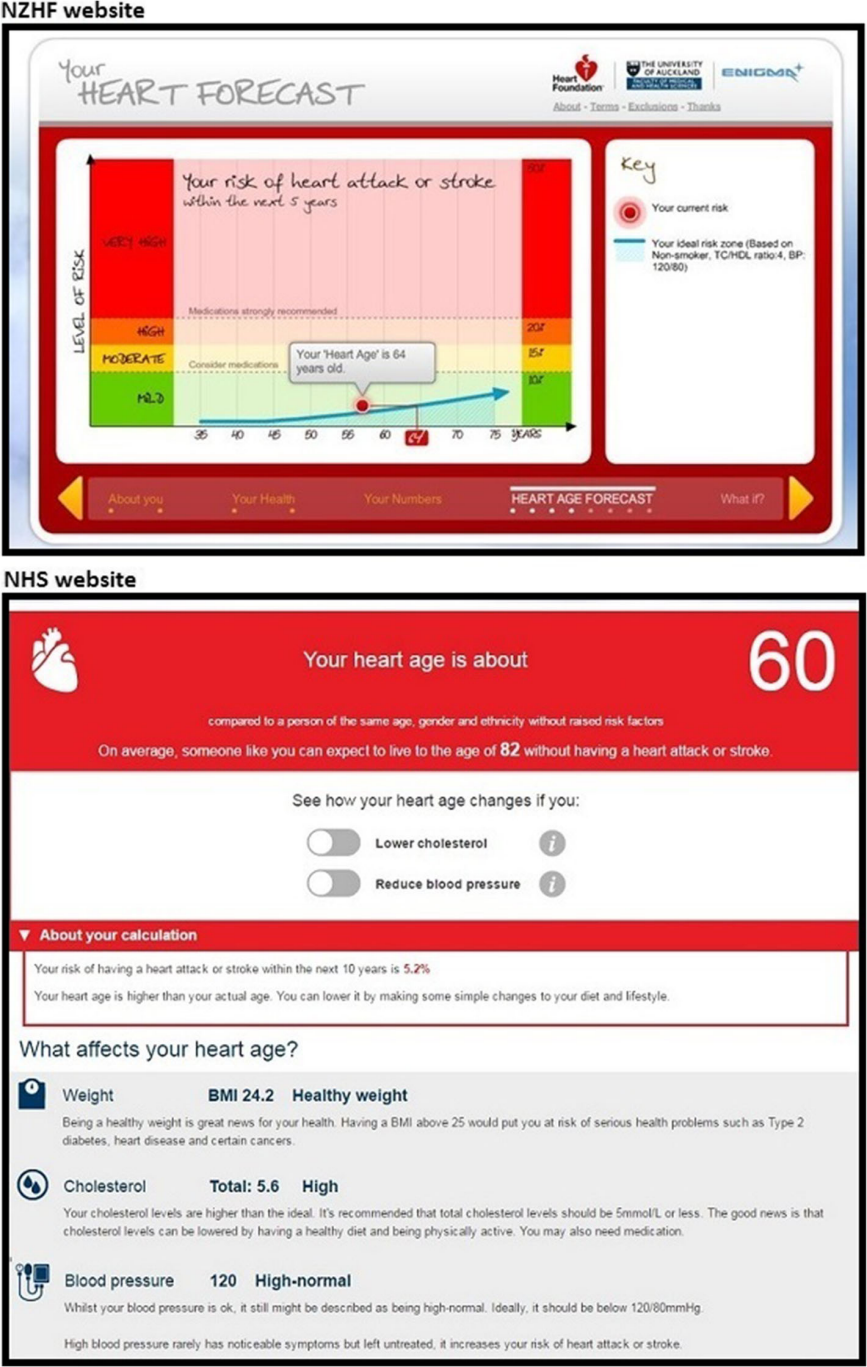
Two heart age calculator results for low absolute risk but older heart age (for Case 1 in Table 1: 57 year old woman with elevated cholesterol and no other risk factors; www.knowyournumbers.co.nz; www.nhs.uk/tools/pages/heartage.aspx) (2017 version)

Heart age generally involves an assessment of risk factors (e.g. age, sex, blood pressure, cholesterol, smoking and diabetes status) to estimate an individual’s risk of CVD, which is then compared to a defined ‘ideal’ [11]. A heart age that is older than current age indicates elevated but modifiable risk, even if the absolute risk of a CVD event in the next 5-10 years is low [5, 11, 12]. For example, the New Zealand Heart Foundation (NZHF) assesses absolute risk in the next 5 years, and compares this to the age at which a person would reach the same absolute risk result if they did not smoke, had systolic blood pressure of 120 mmHg and total/HDL cholesterol ratio of 4 [11]. Figure 1 shows how a 57 year old woman with elevated cholesterol would be assessed as having a low 5-year absolute risk of 4%, but an older heart age of 64, since a woman with ‘ideal’ risk factor levels would not reach 4% risk for another 7 years (www.knowyournumbers.co.nz). This ‘ideal’ absolute risk approach is currently used to promote clinical practice guidelines in New Zealand and the UK [5, 11] but alternative methods compare a patient’s risk factors to the average of the population [13, 14], or use the results of scans rather than absolute risk models [3].

### Where is heart age used?

While New Zealand and the UK are the countries that most clearly link heart age to clinical medication guidelines [5, 11] millions of heart age assessments have been reported internationally in the academic literature. This includes a large study across 13 countries [15] and population estimates of heart age by government health organisations in the US (Centers for Disease Control and Prevention) [16], UK (National Health Service) [17] and China (National Center for Cardiovascular Disease) [18]. In the US, heart age calculators are being promoted by the federal government, local health organisations and industry, though guidelines do not yet appear to explicitly link them to medication recommendations. The Framingham study published a heart age assessment algorithm in 2008 [19], which was developed into an online tool on the Centers for Disease Control and Prevention (CDC) website [20]. This calculator is promoted by the current Million Hearts campaign (federally run through the US Department of Health and Human Services, co-led by the CDC and the Centers for Medicare & Medicaid Services) as a resource for doctors to use with their patients [21]. The CDC published a national estimate of 69.1 million “older” heart age assessments based on this algorithm in 2015 [16], highlighting socio-economic and racial disparities which received some news coverage [2]. Heart age calculators are currently promoted by smaller organisations using various models and presentation formats, including university institutions, medical clinics and private companies [22–24]. More broadly, in 2009 Unilever partnered with the World Heart Federation to promote its heart age calculator and cholesterol-lowering food products internationally, leading to top Google rankings for “heart age” and at least 2.7 million users [15, 25]. In Europe, vascular age for the SCORE model was published in 2010 [26], and a cardiovascular risk age calculator is recommended to communicate the need for lifestyle change to younger adults in 2016 cardiovascular prevention guidelines [27].

### How does heart age fit into CVD risk management?

There are two distinct, and complementary, ways to manage CVD risk: lifestyle change to improve diet and physical activity, and medication to lower blood pressure and cholesterol. Motivating patients with CVD risk factors to change their lifestyle is important at any age, and this is where the heart age concept has been promoted as a potentially useful tool [5, 11, 12]. For example, a 25-year-old obese smoker needs to be motivated to give up smoking and improve their diet and exercise. Their chance of having a heart attack in the next few years will be low due to their young age, so telling them that their heart age is 35 may be a more compelling way to convey the need for lifestyle change. If they still have CVD risk factors at the age of 40, a different approach is needed to make decisions about the benefits of commencing blood pressure and cholesterol-lowering medication. This requires an assessment of the absolute risk of a CVD event (e.g. heart attack or stroke) in the next 5 to 10 years, in order to estimate the benefit an individual patient is likely to gain from taking medication [28].

Problems may arise if heart age is used to inform medication decisions rather than motivate lifestyle change. This is increasingly likely as more heart age assessment methods are developed and implemented alongside medication guidelines based on absolute risk [3, 5, 11]. There are now multiple heart age calculators linked to clinical practice guidelines and available to the public, conveying conflicting messages about risk and medication. It is essential that doctors and patients understand the assumptions behind these heart age calculators and how they relate to absolute risk-based medication guidelines.

### Does heart age improve risk communication?

It can be challenging to communicate CVD risk to patients [12, 29]. Heart age has been promoted as a potentially useful way to explain lifetime CVD risk, particularly for younger people who need to change their lifestyle but are at low risk of a CVD event in the next few years [5, 11]. Clinical trials have shown that paper-based and online risk assessments that include heart age can be beneficial and improve risk factor management compared to standard care with verbal counselling about absolute risk [13, 14, 30]. However, these trials have not directly compared heart age to absolute risk in the same visual format, so although we can say that communicating heart age alongside other CVD risk information can have an effect, we can’t say whether that is due to using heart age instead of absolute risk. Direct experimental comparisons in the general population have found a recall benefit and more emotional impact (e.g. increased worry) of heart age compared to absolute risk, but no advantage for motivating lifestyle change [4, 31, 32]. One of these studies found increased patient misunderstandings about risk level and concerns about credibility in the heart age group [4]. This suggests a need for doctors to explain heart age within the consultation to ensure patients adequately understand their risk and its implications.

**Fig. 2:**
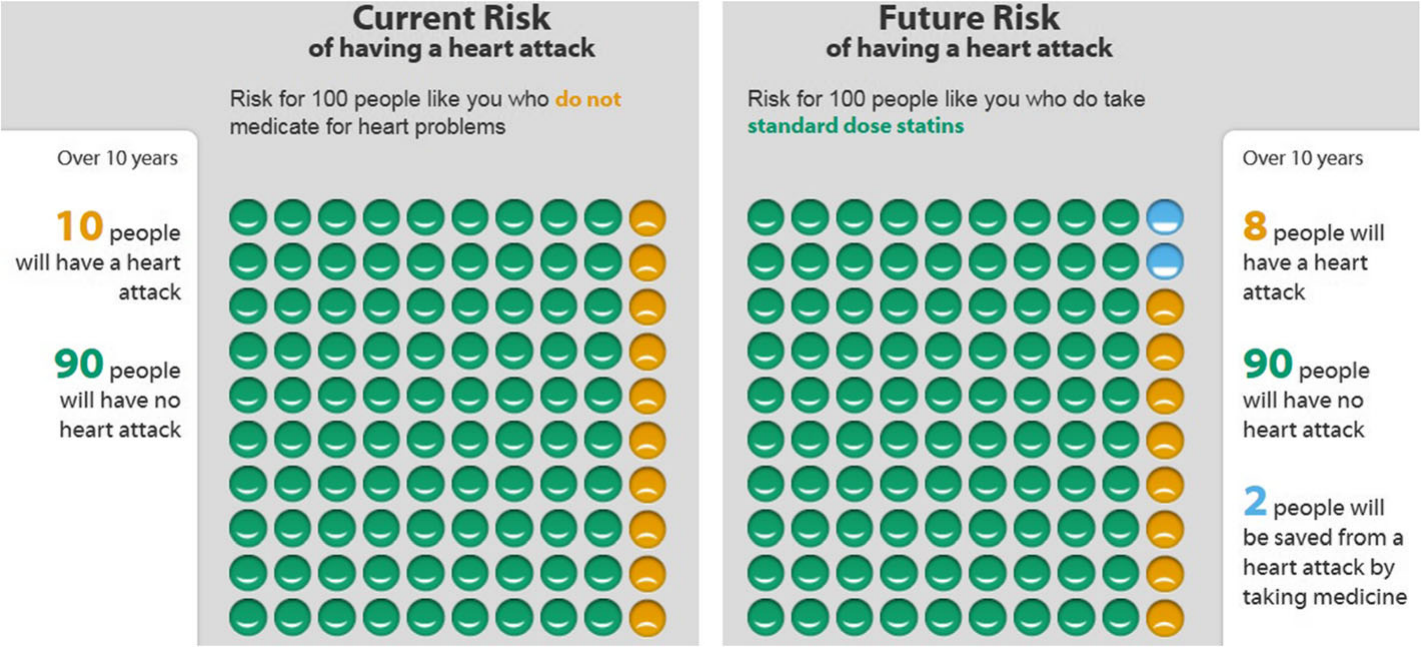
Example of an absolute CVD risk tool from the Mayo Clinic Statin Choice decision aid. (for 10% risk of a CVD event over the next 10 years; https://statindecisionaid.mayoclinic.org/) (2017 version)

Overall, the research suggests that heart age is a more emotionally engaging format for communicating CVD risk to patients, and visual heart age formats may improve risk factor management compared to standard care involving verbal explanations of absolute risk. However, discussions about medication need to be based on absolute risk rather than heart age, because the likelihood of benefit depends on the likelihood of risk. To make an informed decision about medication, patients need to understand their baseline absolute risk, because the probability of preventing CVD with treatment is directly proportional to this [33, 34]. For example, 100 asymptomatic people with a 10-year absolute risk of 10% would need to take statins for 10 years in order to prevent 2 CVD events; the other 98 people would not benefit (90 would not have an event and 8 would have an event despite treatment). Not everyone will think it is worth the cost, inconvenience and side effects to reduce their individual risk from 10% to 8% (see Fig. 2). In this context a shared decision making approach is particularly important, which requires clear communication about the absolute risk of a CVD event, and the absolute benefit of medication [6, 10, 35]. Heart age may help doctors to convey the need for lifestyle change at any level of absolute risk, but it cannot help patients make an informed choice about medication. Further research is needed to investigate the effect of different risk calculation and presentation methods: single versus multiple risk formats (such as combining the percentage with verbal risk level and graphs showing frequency), comparison to average versus ideal risk factors, and use of absolute risk versus scan-based methods of heart age calculation [3].

### Why do heart age calculators give different recommendations for the same patient?

The relationship between heart age and absolute risk thresholds for medication can be very variable [5, 11]. Figure 1 demonstrates two different approaches. The NZHF calculator (www.knowyournumbers.co.nz) explains the relationship between absolute risk and heart age, with medication thresholds based on 5-year absolute risk in line with clinical guidelines. Maintaining ‘ideal’ risk factor levels means that you stay below the medication threshold even with increasing age. The Joint British Societies (JBS) and National Health Service (NHS) heart age calculators (www.jbs3risk.com/pages/risk_calculator.htm; www.nhs.uk/tools/pages/heartage.aspx) estimate 10-year absolute risk, with a separate calculation for heart age based on an annual rate derived from the QRISK lifetime model [4]. The approach to medication is quite different: users are informed that they may need medication based on older heart age, even if their 10-year absolute risk is low. The message conveyed here is that medication is an option regardless of the absolute risk and benefit, which is contrary to the absolute risk approach used in CVD prevention guidelines.

To demonstrate this, we entered real patients’ risk factor values from a previous study of the NZHF calculator into the 2017 version of the NHS calculator [36]. The examples in Table 1 show how people with isolated risk factors but low absolute risk would receive different heart age results and medication information depending on whether they use the NZHF or NHS calculator. For example, Case 1 is a 57 year old non-smoking woman with elevated cholesterol but ‘ideal’ blood pressure, body mass index (BMI) in the healthy range, and no other risk factors. She would receive an older heart age estimate on both the NZHF (64) and NHS (60) websites, but a low absolute risk result of 4% over 5 years or 5% over 10 years. Both the heart age and absolute risk numbers vary due to different underlying models, but the key issue to note for this paper is how “older heart age” is related to medication recommendations. NZHF describes the risk category as mild and below the medication threshold based on absolute risk, while the NHS calculator suggests that both cholesterol and blood pressure medication may be needed, even though the absolute risk is low in both cases. In contrast, if we entered the same risk factors into the American College of Cardiology/American Heart Association (ACC/AHA) absolute risk calculator within the Statin Choice decision aid (see Fig. 2) [37], we would find that 100 people need to take cholesterol medication for 10 years to prevent 1 heart attack, 3 would have a heart attack anyway, and the remaining 96 would never have had a heart attack in the first place. Heart age cannot convey this information.

**Table 1:** Examples of different heart age results for the same patient using different calculators

Patient profile	Risk factors	NZ: HF website	UK: NHS website
*Case 1 with elevated cholesterol but ‘ideal’ blood pressure would receive an older heart age estimate on both calculators, with two medications suggested for the lower result*	Age: 57 Sex: female Systolic BP: 120 Chol ratio: 5.6 BMI: 24 Smoking: no Diabetes: no	Older heart age (64) 5 yr. absolute risk = 4% **Mild risk below medication threshold**	Older heart age (60) 10 yr. absolute risk = 5% **May need chol & blood pressure medication**
*Case 2 with elevated blood pressure but lower than ‘ideal’ cholesterol would receive an older heart age on NZHF or the same heart age as current age on NHS, with one medication suggested for the lower result*	Age: 62 Sex: male Systolic BP: 130 Chol ratio: 3.5 BMI: 25 Smoking: no Diabetes: no	Older heart age (63) 5 yr. absolute risk = 7% **Mild risk below medication threshold**	Same heart age (62) 10 yr. absolute risk = 9% **May need blood pressure medication**
*Case 3 with obesity but ‘ideal’ blood pressure and cholesterol would receive a younger heart age on NZHF or an older heart age on NHS, with one medication suggested for the higher result*	Age: 48 Sex: female Systolic BP: 120 Chol ratio: 4 BMI: 38 Smoking: no Diabetes: no	Young heart age (< 48) 5 yr. absolute risk = 1% **Mild risk below medication threshold**	Older heart age (49) 10 yr. absolute risk = 2% **May need blood pressure medication**

*Chol ratio* total/HDL cholesterol ratio, *BMI* body mass index, *NZ HF* New Zealand Heart Foundation website (www.knowyournumbers.co.nz) (2017 version), *UK NHS* United Kingdom National Health Service website (www.nhs.uk/tools/pages/heartage.aspx) (2017 version)

As well as using different CVD risk models, risk factor thresholds may differ between calculators. Heart age is based on comparison to an ‘ideal’, which requires the use of multiple thresholds for individual risk factors. For example, Case 1 is right on the threshold for blood pressure (120 mmHg) so she is given the message that her CVD risk is higher than normal on the NHS website; whereas this is described as ‘ideal’ on the NZHF website (2017 versions). The NHS website also says that cholesterol medication may be needed despite being well under the absolute risk medication threshold of 10%. The heart age results in Table 1 are not widely different in absolute terms, but there is a psychological difference between having a younger and older heart age [36].

The examples above show how different heart age calculators can lead to different medication recommendations. This suggests that patients with the same risk factors may perceive their risk differently and receive different treatment recommendations depending on which calculator their doctor uses; potentially leading to unwarranted practice variation. These differences will become even greater when using average rather than ideal risk factors [13, 14], or scan-based methods of calculation rather than absolute risk [3].

### How does heart age relate to medication decision making?

Using heart age to recommend medication is likely to undermine the absolute risk approach for medication decisions. The use of absolute risk to make treatment decisions, instead of treating blood pressure and cholesterol as isolated risk factors, allows medication to be targeted to those at highest risk who are most likely to benefit by prevention of a CVD event within 5-10 years. This approach aims to prevent both over-treatment of low risk and under-treatment of high risk individuals [28].

However, while JBS3 guidelines focus on absolute risk assessment, they also recommend that medication should be considered for people with low absolute risk but older heart age than current age [5]. This is likely to further lower the threshold for medication, and lead to over-treatment of low risk individuals. National estimates of heart age by government health organisations in the US, UK and China show how this approach could lead to ‘mass medicalisation’, as the majority of the general population has an older heart age than current age. The CDC found that every age strata had an older heart age than current age on average, and 69.1 million (43.7%) people aged 30-74 had a heart age > 5 years older than current age [16]. The first 1.4 million users of the UK National Health Service heart age tool indicated that 79% had an older heart age than current age in every age strata, including 69% of users under 40 years, who are at low absolute risk of CVD [17]. The China National Centre for Cardiovascular Disease analysed heart age assessments for 18,214 people and found a mean heart age 10 years older than current age, despite a low 10-year CVD risk of 4% [18]. Similarly, an international study of 2.7 million people (31% UK) found an average heart age 4 years older than current age [15].

Other risk format suggestions in the cardiovascular literature include relative risk, lifetime risk and percentiles [12], but they all have the same problem as heart age: they do not enable patients to make an informed choice about medication benefits and harms based on current research evidence. The 2016 European guidelines make a clearer distinction between absolute risk for medication and heart age for lifestyle, stating directly: “both risk age and lifetime risk are closer to relative than absolute risk, and none provides an evidence base for drug treatment decisions” [27]. Table 2 demonstrates how to avoid the potential harms of using heart age to justify medication.

**Table 2:** Why caution should be used when linking heart age to medication

• **Practice variation:** Heart age results may be younger, the same or older than current age for the same risk factors, depending on the CVD risk model and ideal risk factor thresholds used to calculate heart age; so it is important for doctors to understand the assumptions behind these calculators in order to avoid unwarranted practice variation.
• **Uninformed decision making:** Patients cannot understand the chance of benefiting from preventive CVD medications such as statins and blood pressure lowering drugs without knowing the baseline absolute risk; so the relationship between heart age, absolute risk and recommended medication thresholds needs to be explained to enable informed decision making.
• **Over-treatment:** Using heart age to decide on the need for drugs will lead to treatment of people who are very unlikely to experience a CVD event in the next 5-10 years; so treatment decisions should be made on the basis of absolute risk and not heart age.

### What could we use instead of heart age?

The risk communication and decision aid literature provides clear directions for alternative absolute risk formats that could be used to explain CVD risk and the benefits of both lifestyle and medication interventions. This should be based on outcomes that are meaningful to patients, focusing on the likelihood of experiencing a CVD event rather than how far blood pressure or cholesterol deviates from an arbitrary ‘ideal’ threshold [6, 10, 35]. We know that the absolute risk of a CVD event is easier to understand than relative risk, and that multiple formats will cover different patient information preferences and learning styles [38]. This should include verbal explanation of the frequency of CVD events for a given absolute risk result, and visual formats showing the risks and benefits of all lifestyle and medication options in absolute terms [38]. Recent qualitative research investigating such visual formats demonstrated how patients find absolute risk more meaningful when both lifestyle and medication intervention effects can be explored in relation to this (e.g. using the risk calculator at http://chd.bestsciencemedicine.com/calc2.html) (2017 version) [39]. There is strong evidence from a Cochrane systematic review of 105 randomised controlled trials that providing this sort of information in the form of a patient decision aid improves patient knowledge, accuracy of risk perception, doctor-patient communication, and decision making that is consistent with individual values and preferences [40]. For example, the Statin Choice decision aid is an evidence-based, effective tool that demonstrates how this can be done (Fig. 2), but it could be improved by allowing lifestyle interventions to be compared to medication options [37].

In the context of CVD prevention, where the majority of asymptomatic people may be told they are high risk based on heart age assessment, but the likelihood of benefiting from medication depends on absolute risk, clear communication to enable shared decision making is especially important [41]. This requires population-based absolute risk and treatment efficacy data to be provided to the patient in a transparent way.

*“When we offer statins, or any preventive treatment, we are practicing a new kind of medicine, very different to the doctor treating a head injury in A&E. We are less like doctors, and more like a life insurance sales team: offering occasional benefits, many years from now, in exchange for small ongoing costs. Patients differ in what they want to pay now, in side effects or inconvenience, and how much they care about abstract future benefits. Crucially, the benefits and disadvantages are so closely balanced that these individual differences really matter.” (Ben Goldacre, BMJ 2014)* [10].

## Conclusions

The heart age concept has intuitive appeal, but it can also be used to justify treatment of low risk people who are very unlikely to benefit in the short term from taking medication. The assumptions behind heart age calculators need to be made clear and explicit so that both doctors and patients can understand why they may get different results on different calculators. They also need to understand that this is a relative measure, where an individual is compared to a specific definition of the ‘ideal’. Informed medication decisions should be based on absolute risk and benefit rather than heart age, and evidence-based communication formats such as decision aids should be used to avoid uninformed decision making and overtreatment of healthy, asymptomatic people who are at low risk of a heart attack or stroke.

## Abbreviations

A&E: Accident & Emergency Department
ACC/AHA: American College of Cardiology/American Heart Association
BMI: Body mass index
BMJ: British Medical Journal
CDC: Centers for Disease Control and Prevention
CVD: Cardiovascular disease
HDL: High-density lipoprotein
JBS: Joint British Societies
NHS: National Health Service
NZHF: New Zealand Heart Foundation
UK: United Kingdom
US: United States of America
